# Social isolation and health risks: Do the younger generations behave differently? Findings from the European Social Survey (Round 11 – 2023/2024)

**DOI:** 10.1371/journal.pone.0357725

**Published:** 2026-09-10

**Authors:** Carla Blázquez-Fernández, Paloma Lanza-León, David Cantarero-Prieto

**Affiliations:** 1 Department of Economics, Universidad de Cantabria, Santander, Cantabria, Spain; 2 Health Economics Research Group, Valdecilla Health Research Institute (IDIVAL), Santander, Cantabria, Spain; 3 Santander Financial Institute, SANFI, Santander, Cantabria, Spain; Atal Bihari Vajpayee Institute of Medical Sciences & Dr Ram Manohar Lohia Hospital, INDIA

## Abstract

Social isolation has long been recognized as a determinant of poor health, particularly in older adults. However, its effects on young people remain underexplored. This study investigates how different dimensions of social isolation affect health-related behaviors among different generations (Silent Generation, Baby Boomers, Generation X, Millennials, and Generation Z), including physical inactivity, tobacco and alcohol consumption, and body mass index as dependent variables. Using cross-sectional data from Round 11 of the European Social Survey, we analyze a sample of individuals from 24 European countries. We apply logistic regression models to estimate the association between five social isolation indicators (non-social meetings, lack of participation in activities, living alone, lack of emotional support, and unhappiness) and the above-mentioned four health-related outcomes. The empirical results highlight that social isolation is meaningfully associated with health behaviors, even among younger cohorts (e.g.: non-social meetings [OR = 1.291–1.789] play a key role in contributing to physical inactivity across all generations). It could be said that Generation X represents a transitional group, balancing traditional and modern lifestyles. Our findings emphasize the need for age-specific policies to address social isolation and promote lifelong health and support urgent policy attention to mitigate the long-term public health consequences of social disconnection in Europe.

## Introduction

In contemporary societies, single-person households (without children) have exhibited an increase in recent years within the European Union, rising by 16.90% from 2015 to 2024, compared to 5.8% for all households [1]. Consequently, social isolation has become a prevalent phenomenon within society over time. However, the question arises as to whether living alone is synonymous with social isolation or loneliness. Individuals can experience social isolation without necessarily feeling lonely, or conversely, feel lonely despite possessing a sufficient number of social relationships [2]. However, there is still no clear consensus in the literature on this matter. Some researchers consider social isolation and loneliness as two different but connected concepts [3], with social isolation being more directly associated with the quantity of an individual’s social interactions and contacts rather than the quality of these relationships. Conversely, other authors make no distinction between the two terms, considering loneliness a subjective measure of perceived isolation in the absence of an objective metric for social integration and interaction [4]. Social isolation can be classified into different types: emotional isolation, which implies the absence of close emotional bonds; structural isolation, involving a lack of integration into existing social networks and the absence of reciprocal friendships; and social loneliness, focusing on the absence of broader social connections [5]. Thus, while loneliness represents a subjective feeling, social isolation constitutes an objective measure of connections [4,6], often indicated by factors such as living alone, marital status, a reduced social network, infrequent participation in social activities, limited social contacts or feelings of loneliness and lack of support.

Integration within social groups is a fundamental determinant of psychological and behavioral health [7,8]. Consequently, social isolation represents a significant concern from a public health perspective, exerting a detrimental impact on health outcomes [9,10]. Furthermore, it poses significant social challenges across all ages, being both a concern and a risk factor for poor health [11] and adverse lifestyle behaviors, including obesity, alcohol consumption, and tobacco use [9,12]. The prevalence of social isolation has been exacerbated by the 2019 coronavirus disease pandemic and its associated social distancing measures.

Social isolation is a phenomenon present across all age groups, demonstrating a consistent increase with ageing [13]. Nevertheless, younger adults may experience the effects of social isolation differently compared to older adults [9]. Indeed, early adulthood is characterized by distinct psychosocial challenges, such as the assumption of greater responsibilities and focus on social connections (friendships and romantic partnerships, etc.) other than family relationships [7]. Despite the predominant focus of research analyzing the health risks associated with social isolation in late adulthood or old age [14,15], attention should also be directed towards young and middle-aged adults. The existing body of literature concerning socially isolated youth (distinct from physical isolation, as experienced during the COVID-19 pandemic) remains limited and often concentrates on specific behaviors, such as alcohol or tobacco consumption [16]. While much of the literature focuses on the role of social isolation in health behaviors, related work in the economics domain illustrates the broader influence of social networks on life outcomes. Kalfa and Piracha [17] show that social capital and personal networks can exacerbate labor-market mismatches, highlighting that social structures exert heterogeneous effects on individual outcomes across contexts.

Considering the existing evidence, this study aims to investigate the association between social isolation and specific health-related factors, with a particular emphasis on healthy lifestyle behaviors (physical activity and body mass index (BMI)) and the consumption of certain substances (tobacco and alcohol) within the European population. To further this objective, this research seeks to examine whether the associations between social isolation and health-related behaviors vary across different age cohorts, with a primary focus on young adults. This objective posits the hypothesis that greater social isolation will be associated with poorer outcomes in health-related factors among Europeans. Our study contributes to evidence by demonstrating that even in young people, social isolation is strongly associated with unhealthy lifestyles – to a degree that in some cases exceeds that observed in older groups. Recent evidence further supports the idea that the effects of social isolation on health behaviors are not uniform across age groups, concluding that social isolation has significant and heterogeneous impacts on health and socioeconomic outcomes across European generations [18].

## Literature review

In light of the social challenges posed by isolation and its significant implications for health, empirical evidence has explored the relationship between social isolation and its effects on various health-related factors and behaviors [2,9,14]. Empirical research highlights that the detrimental impact of social isolation on health is mediated, to a large extent, by behavioral mechanisms, particularly during younger stages of life [10,13].

Physical activity is consistently identified as a protective factor for affective well-being. Isolated individuals are more likely to exhibit an unhealthy lifestyle [13] and less likely to practice regular physical activity [11]. Benedyk et al. [19] demonstrate that young adults experiencing affective distress due to social disconnection report improved emotional states when they practice physical exercise. Importantly, the type of isolation (social avoidance, active isolation, and social indifference) significantly influences adolescents’ physical activity patterns [20]. Furthermore, physical inactivity appears to be one of the mediating factors in the relationship between isolation and increased mortality [21].

The relationship between isolation and substance use is complex and age-dependent. Adolescents may exhibit differential engagement in healthy behaviors depending on their experience of isolation and their position within a social group. While some socially isolated are more susceptible to detrimental habits, such as smoking or alcohol consumption, to manage anxiety and loneliness [2], others are less exposed to substance-using peer groups, and isolation could serve as a protective factor against such consumption [22]. Niño et al. [16] show that socially indifferent adolescents are more likely to consume alcohol and tobacco, whereas those who actively avoid social contact show lower risk. In adulthood, isolation is linked with elevated risk of substance use disorders. Desai et al. [23] reported that socially isolated hospitalized patients frequently used tobacco, alcohol, cannabis, and opioids.

Similarly, social isolation is associated with increased alcohol consumption, particularly among young adults [24]. Segrin et al. [25] demonstrate that negative emotional traits manifested in neuroticism, depression, anxiety, and stress sensitivity indirectly contribute to solitary drinking through heightened social isolation. During the COVID-19 pandemic, socially isolated drinkers exhibited a greater increase in negative effect than non-drinkers [26].

Social isolation is also linked to poor dietary habits and nutritional outcomes, characterized by low fruit and vegetable intake and reduced diet quality. Socially isolated individuals often consume fewer fruits, vegetables, and fish, leading to reduced diet quality [2,13,27]. Social isolation is associated with both undernutrition and food insecurity, defined as limited access to foods necessary for an active and healthy life, especially among older and middle-aged adults [28–30]. These effects are particularly pronounced in individuals who are also obese, representing a vulnerable group [31]. In a psychological perspective, Tomova et al. [32] found that acute isolation leads to social craving in a manner analogous to hunger as a response to food deprivation. During the COVID-19 lockdowns, young people reported increases in isolation, pointing to their vulnerability and the long-term implications for lifestyle and mental health [33].

The impact of social isolation is uneven across generations, shaped by technological fluency, social norms, and life course stages. Members of the Silent Generation (born before 1946) are particularly vulnerable to isolation due to functional limitations and lower levels of digital literacy [34]. In terms of health behaviors, Ross and de Jager [35] detail how loneliness among older women is experienced emotionally and socially, often leading to reduced physical activity and poorer dietary habits. In addition, technology-related inequalities may further aggravate sedentary behavior or increased substance use due to isolation [7].

Although more digitally connected, Baby Boomers (born 1946–1964) remain vulnerable to isolation due to widowhood, retirement, or living alone. They represent a transitional generation, balancing traditional aging with increasing technological engagement. Demey et al. [36] show that pathways into living alone in mid-life are diverse but frequently lead to social disconnection. Hawkley et al. [37] identify cohort-specific trends in loneliness, finding that Baby Boomers are experiencing rising levels of social isolation over time.

Generation X (born 1965–1980) balances digital literacy with high levels of professional and familial stress. Lissitsa and Kagan [38] find that self-reliance moderates the link between loneliness and social media addiction among men in Generations X, Millennials, and Z, suggesting that greater self-reliance may protect against the negative effects of isolation on digital well-being. In a related study, Lissitsa and Kagan [39] show that childhood bullying leads to increased loneliness and social media addiction in adulthood, with self-esteem playing a key mediating role across these generations.

Millennials (born 1981–1996) have experienced the effects of digital transition, economic instability, and pandemic-induced isolation [33]. Their health behaviors reflect this complexity: while they show declining alcohol use partly linked to “generation sensible” health-oriented norms and individualization [40], their experiences of isolation have intensified derived from the social distancing policies [41]. Some substitute in-person connection with virtual spaces like the metaverse, which can either mitigate loneliness [42].

As the most digitally immersed cohort, Generation Z (born after 1996) faces high levels of social isolation, anxiety, and hyperconnectivity-induced stress. Digital surveillance, hyperconnectivity and shifting communication norms exacerbate their vulnerability [43,44]. Emotional intelligence may buffer some of these effects in the workplace [45], but many still require targeted psychological support in educational and professional contexts [8,46]. These patterns may contribute to unhealthy lifestyle habits, including reduced physical activity, emotional eating, or increased substance experimentation.

## Methodology and data

### Data

Basic data used in this study is taken from the European Social Survey (ESS). The ESS is an international comparative study conducted every two years since 2001 through different European countries. Indeed, 39 countries have participated in at least one round since 2002–2003, whereas 31 countries participated in ESS Round 11 (latest available when doing this paper). Its main objective is to collect data on the attitudes, beliefs, and behavioral patterns of European populations. The survey is conducted through face-to-face interviews, and its questionnaire includes a fixed core module and rotating modules that address specific topics in each edition [47].

In this study, cross-sectional data is applied. Precisely data from the Round 11 Survey, for years 2023–2024 (latest available one), is used through 24 European countries. ESS non-substantive response categories, including ‘Refusal’, ‘Don’t know’, and ‘No answer’, were coded as missing values. The empirical analysis was conducted using complete cases only; therefore, respondents with missing information in any of the variables included in each model were excluded from the corresponding regression analysis. Moreover, macro-region clusters, according to EuroVoc (Europe is divided into four subregions: Central and Eastern, Northern, Southern, and Western Europe), are used in our estimates. However, since interest variables are not in all the rounds, non-panel data techniques could be applied here.

### Variables

Because our objective is to analyze the health risks associated with social isolation, dataset covers, following the above-mentioned studies included in the literature review, as main explanatory variables five “social isolation factors” (*non_social_meeting*, *non_social_act*, *living_alone*, *non_emotional_support*, and *unhappy*) and four population-level health risk factors (*physical_inactivity*, *smoker*, *frequent_drinker*, and *obese_overweight*) as dependent ones. Additionally, other variables associated with both socio-economic and health care factors are used as controls. Table 1 presents the details concerning the description of the variables whereas Table 2 contains the basic descriptive statistics.

**Table 1:** Variables used, description and coding.

VARIABLE		DESCRIPTION	CODING
***Health risks factors: DEPENDENT VARIABLES***	*physical_inactivity*	Physical inactivity	1: if the respondent walks quickly, do sports or other physical activity for 30 minutes or longer fewer than three days a week; 0: otherwise
	*smoker*	Smoker	1: if the respondent smoke (regardless the number of cigarettes); 0: otherwise
	*frequent_drinker*	Frequent drinker	1: if respondent indicate drinking alcohol “every day” or “several times a week”; 0: otherwise
	*obese_overweight*	Obese or overweight	1: if BMI (Body Mass Index), calculated from self-reported height and weight, is ≥ 25 kg/m²; 0 otherwise
***Generation***	*silent_generation*	Silent generation	1: if the individual is born between 1928 and 1945; 0: otherwise
	*baby_boomers*	Baby boomers	1: if the individual is born between 1946 and 1964; 0: otherwise
	*generation_x*	Generation X	1: if the individual is born between 1965 and 1980; 0: otherwise
	*millennials*	Millennials	1: if the individual is born between 1981 and 1996; 0: otherwise
	*generation_z (reference)*	Generation Z	1: if the individual is born between 1997 and 2010; 0: otherwise
***Socio-economic*** ***factors***	*female*	Gender of respondent	1: if female; 0: if male
	*low_education (Reference)*	ISCED-2011 coding of education, levels 0–2	1: if low education, levels 9−2; 0: otherwise
	*middle_education*	ISCED-2011 coding of education, levels 3–4	1: if middle education, levels 3–4; 0: otherwise
	*high_education*	ISCED-2011 coding of education, levels 5–8	1: if high education, levels 5–8; 0: otherwise
	*rural*	Area of location (place of residence)	1: if respondent lives in country village, farm or home in countryside; 0: otherwise
	*low_income (Reference)*	Low income, deciles 1–3	1: if person is in these income deciles, 1–3; 0: otherwise
	*middle_income*	Middle income, deciles 4–7	1: if person is in these income deciles, 4–7; 0: otherwise
	*high_income*	High income, deciles 8–10	1: if person is in these income deciles, 8–10; 0: otherwise
***Health factors***	*sah_b_vb*	Self-Assessed Health (SAH) bad or very bad	1: if SAH bad or very bad; 0: otherwise
	*disabled*	Disabled	1: if the respondent is hampered in its daily activities in any way by any longstanding illness, or disability, infirmity or mental health problem; 0: otherwise
***Social isolation factors***	*non_social_meeting*	Non-social meetings	1: if the individual reports social meeting with friends, relatives or colleagues less than once a month or never; 0: otherwise
	*non_social_act*	Non-social activities	1: if the individual takes part in social activities less than most or much less than most compared to others of same age; 0: otherwise
	*living_alone*	Living alone	1: if the respondent lives alone; 0: otherwise
	*non_emotional_support*	Non-emotional support	1: if the individual reports having no one with whom they can discuss intimate and personal matters; 0: otherwise
	*unhappy*	Unhappy	1: if the respondent reports a happiness score between 0 and 4 on the ESS 0–10 happiness scale, where 0 means ‘Extremely unhappy’ and 10 means ‘Extremely happy’; 0 otherwise
***Macro-regional factors***	*Southern (Reference)*	Regions of Europe according to EuroVoc. Country of the respondent is in Southern Europe	1: if country is Cyprus, Spain, Greece, Italy, or Portugal; 0: otherwise
	*Northern*	Regions of Europe according to EuroVoc. Country of the respondent is in Northern Europe	1: if country is Finland, Iceland, Lithuania, Norway, or Sweden; 0: otherwise
	*Western*	Regions of Europe according to EuroVoc. Country of the respondent is in Western Europe	1: if country is Austria, Belgium, Switzerland, Germany, France, United Kingdom, Ireland, or the Netherlands; 0: otherwise
	*Central_and_Eastern*	Regions of Europe according to EuroVoc. Country of the respondent if in Central or Eastern Europe	1: if country is Croatia, Hungary, Poland, Serbia, Slovenia, or Slovakia; 0: otherwise

Source: Authors’ elaboration.

**Table 2:** Descriptive statistics: distribution of the analytical sample, percentages (%).

VARIABLES		FULL SAMPLE (n = 29,337)	SILENT GENERATION (n = 2,084; 7.10%)	BABY BOOMERS (n = 9,086; 30.97%)	GENERATION X (n = 8,089; 27.57%)	MILLENNIALS (n = 6,906; 23.54%)	GENERATION Z (n = 3,172; 10.81%) -*Reference*
***Health risks factors: DEPENDENT VARIABLES***	*physical_inactivity*	42.36	60.12	45.98	42.16	37.69	30.99
	*smoker*	22.42	5.66	18.53	26.88	27.44	22.29
	*frequent_drinker*	21.53	23.18	26.79	22.54	17.39	11.85
	*obese_overweight*	54.90	56.62	64.48	60.55	46.97	29.19
***Socio-economic*** ***factors***	*female*	52.03	55.81	51.35	57.87	51.88	49.62
	*low_education (Reference)*	20.48	47.60	27.88	13.98	8.34	24.46
	*middle_education*	43.69	30.61	44.14	46.00	40.43	52.18
	*high_education*	35.52	21.02	27.65	39.73	51.02	23.11
	*rural*	40.47	41.89	44.05	42.27	34.61	37.45
	*low_income (Reference)*	25.94	52.93	33.87	18.99	17.00	22.73
	*middle_income*	45.02	38.00	47.96	43.52	45.29	44.48
	*high_income*	29.03	9.07	18.17	37.50	37.71	32.79
***Health factors***	*sah_b_vb*	6.93	19.72	10.10	5.65	2.59	2.18
	*disabled*	26.80	54.03	35.86	23.72	15.55	15.29
***Social isolation factors***	*non_social_meeting*	10.04	19.58	12.60	10.40	6.56	3.53
	*non_social_act*	37.76	47.84	39.72	37.95	34.97	31.09
	*living_alone*	22.45	51.87	28.86	17.12	15.45	13.55
	*non_emotional_support*	4.06	8.73	5.88	3.43	2.09	1.70
	*unhappy*	5.31	8.35	5.87	5.20	4.26	4.26
***Macro-regional factors***	*Southern (Reference)*	20.21	21.26	19.22	21.35	20.10	19.67
	*Northern*	18.81	18.57	18.60	18.69	19.77	18.44
	*Western*	39.26	41.84	39.18	38.29	39.81	39.01
	*Central_and_Eastern*	21.66	18.33	23.00	21.67	20.33	22.82

Source: Authors’ elaboration.

### Statistical analysis

Given the nature of the dependent variables (the four are binary ones, and each one could be denoted by y), discrete choice models are particularly well suited to the analysis. Indeed, in this study, logit models are going to be used (1):


$$\begin{array}{c} p=Prob\;(y=\text{1}|X)=\frac{\text{exp}\left(X^{\prime }\beta \right)}{\text{1}+\text{exp}\left(X^{\prime }\beta \right)} \end{array}$$ (1)

In the logit model, the conditional (to X) probability is described by the cumulative logistic distribution (that is, conditional to some explanatory variables X) being the predicted probabilities always between zero and one. Besides, results are going to be presented through Odds Ratios (OR), to be understood interdisciplinary, and can be expressed as follows (2):


$$\begin{array}{c} \text{ln}\left(\frac{p}{\text{1}-p}\right)=\text{ X}\text{'}\beta \end{array}$$ (2)

Odds are defined as the ratio of the probability of success and the probability of failure. Then, OR = 1 implies that the variable has no effect on the odds of the event. However, if we obtain and OR > 1 would represent that the variable increases the odds of the event happening, whereas an OR < 1 implied that the variable decreases the odds of the event happening.

The correlation matrix was used to identify possible multicollinearity between variables. Based on this matrix, there do not appear to be major multicollinearity issues, and we estimated our models with the abovementioned factors/variables. Given the complex, multi-stage sampling design of the ESS, all regression models were estimated using the ESS analysis weight.

**Fig 1:**
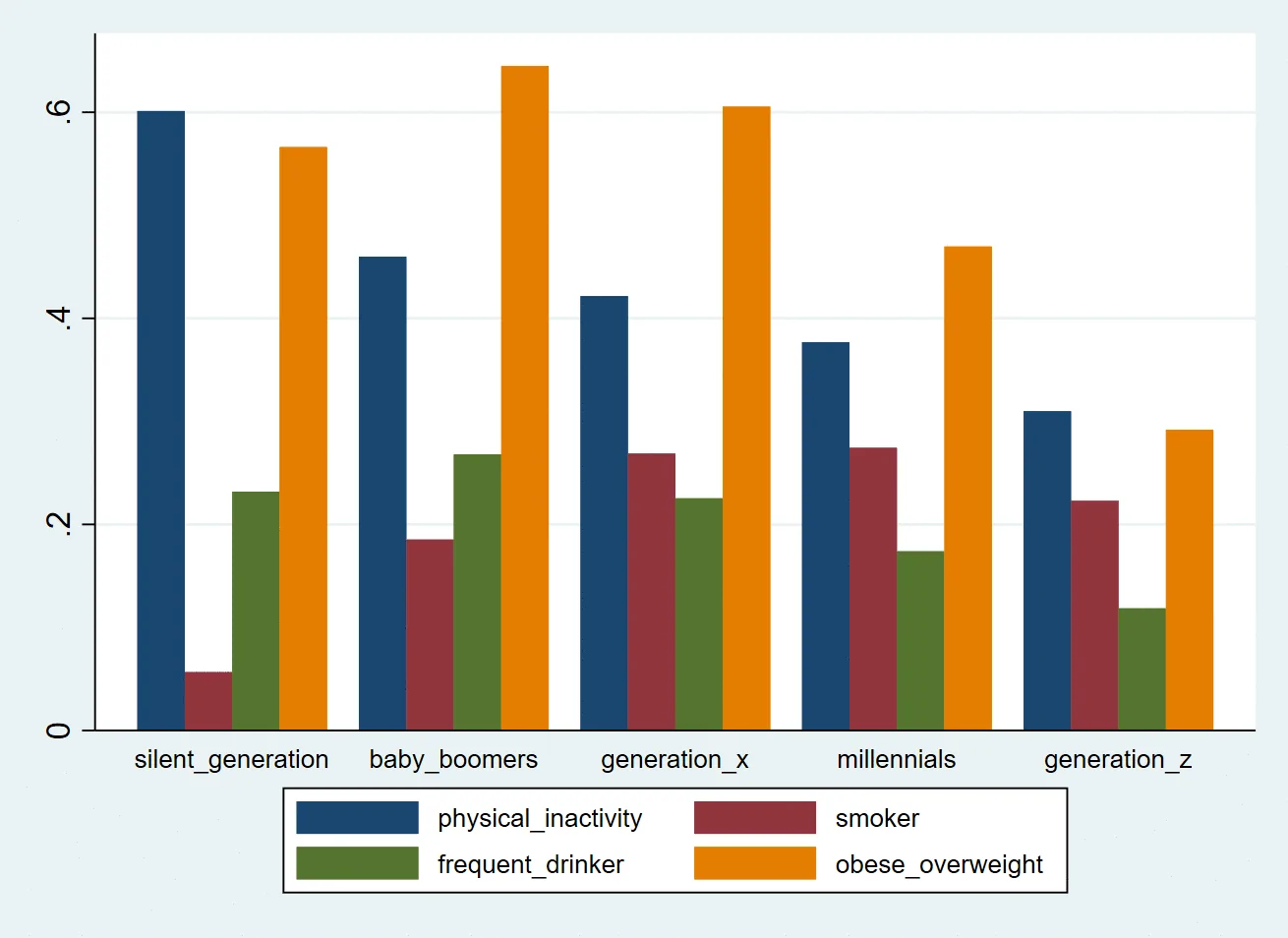
Distribution of health risks factors by generation. Source: Authors’ elaboration.

**Fig 2:**
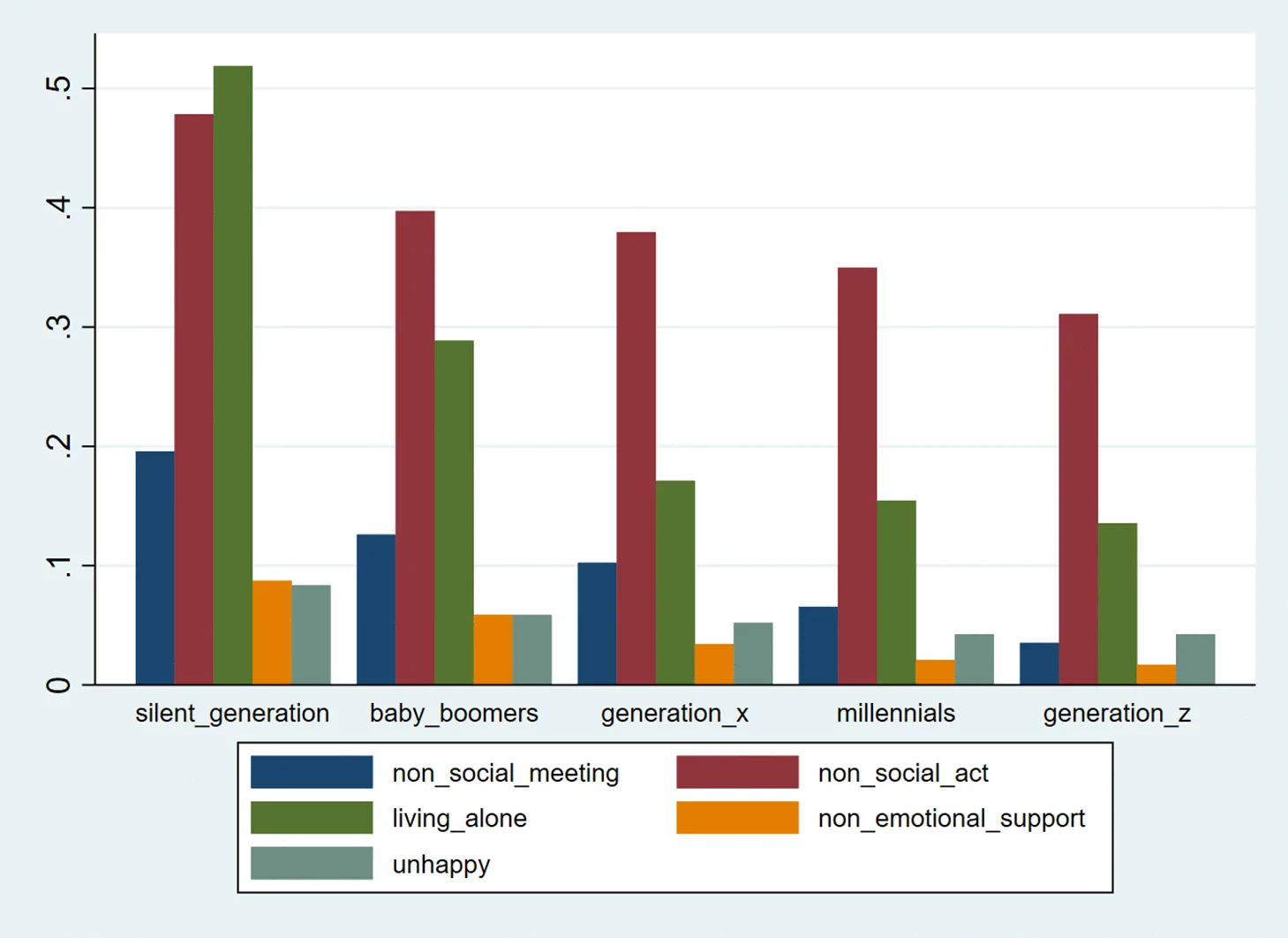
Distribution of social isolation factors by generation. Source: Authors’ elaboration.

All in all, in order to take a look at our data before presenting the empirical results we have done two representative figures. Fig 1, plots the distribution of health risks factors by generation while Fig 2, exemplifies the distribution of social isolation factors by generation.

From these figures, we can observe that there is not a clear pattern and that each generation behaves differently. However, in Fig 1, more similarities can be detected between Generation X, Millennials and Generation Z, where the higher percentages are for *obese_overweight* followed by *physical_inactivity*, *smoker* and *frequent_drinker* whereas Baby Boomers’ *frequent_drinker* is higher than *smoker*. Regarding the Silent Generation, *physical_inactivity* presents the highest results and as for Baby Boomers’ *frequent_drinker* is higher than *smoker*. From Fig 2, it can be highlighted that the higher the age, the higher the social isolation factors that appear.

## Results

Tables 3–6 summarize the empirical results. We have presented in each of the tables the results per dependent variable. Precisely, it contains the OR (95% CI) for both the full sample and considering the disaggregation by generation: Silent Generation, Baby Boomers, Generation X, Millennials, and Generation Z. Thus, Table 3 focuses on *physical_inactivity*, Table 4 is for is for *smoker* whereas *frequent_drinker* is in Table 5. Moreover, Table 6 is devoted to *obese_overweight*.

**Table 3:** Logistic estimates. Dependent variable: *physical_inactivity.*

VARIABLES	FULL SAMPLE (n = 29,337)				SILENT GENERATION (n = 2,084)				BABY BOOMERS (n = 9,086)				GENERATION X (n = 8,089)				MILLENNIALS (n = 6,906)				GENERATION Z (n = 3,172)			
	OR	95%CI			OR	95%CI			OR	95%CI			OR	95%CI			OR	95%CI			OR	95%CI		
*silent_generation*	2.128	[1.677	2.700]	^***^																				
*baby_boomers*	1.584	[1.335	1.879]	^***^																				
*generation_x*	1.514	[1.294	1.772]	^***^																				
*millennials*	1.532	[1.309	1.793]	^***^																				
*generation_z (reference)*																								
*female*	1.071	[0.979	1.172]	^*^	1.387	[0.936	2.055]	^*^	1.107	[0.936	1.310]		0.874	[0.732	1.043]		1.099	[0.925	1.306]		1.286	[0.963	1.718]	^*^
*low_education (reference)*																								
*middle_education*	0.922	[0.812	1.048]		0.922	[0.603	1.409]		0.881	[0.719	1.080]		1.030	[0.807	1.314]		0.995	[0.731	1.355]		1.082	[0.783	1.496]	
*high_education*	0.661	[0.575	0.760]	^***^	0.707	[0.407	1.230]		0.542	[0.423	0.694]	^***^	0.738	[0.581	0.937]	^***^	0.765	[0.550	1.065]	^*^	0.787	[0.546	1.134]	
*rural*	1.043	[0.953	1.142]		1.557	[1.054	2.302]	^**^	1.154	[0.976	1.365]	^*^	0.917	[0.780	1.077]		0.954	[0.794	1.147]		1.114	[0.845	1.468]	
*low_income (reference)*																								
*middle_income*	1.154	[1.020	1.304]	^**^	1.329	[0.887	1.991]		1.239	[1.005	1.526]	^**^	1.235	[0.974	1.565]	^*^	0.935	[0.711	1.229]		1.197	[0.798	1.797]	
*high_income*	1.097	[0.951	1.264]		1.254	[0.574	2.738]		1.175	[0.903	1.529]		1.207	[0.925	1.574]		0.831	[0.630	1.096]		1.024	[0.651	1.613]	
*sah_b_vb*	1.802	[1.460	2.224]	^***^	2.160	[1.193	3.912]	^***^	1.633	[1.197	2.227]	^***^	2.092	[1.420	3.083]	^***^	1.799	[0.961	3.366]	^*^	1.239	[0.546	2.812]	
*disabled*	1.448	[1.287	1.628]	^***^	1.759	[1.222	2.534]	^***^	1.740	[1.442	2.100]	^***^	1.247	[0.985	1.577]	^*^	1.306	[1.015	1.681]	^**^	1.258	[0.810	1.953]	
*non_social_meeting*	1.428	[1.215	1.679]	^***^	1.700	[0.996	2.902]	^**^	1.291	[0.986	1.690]	^*^	1.377	[0.999	1.898]	^**^	1.359	[0.944	1.958]	^*^	1.789	[0.933	3.429]	^*^
*non_social_act*	1.370	[1.246	1.506]	^***^	2.094	[1.453	3.018]	^***^	1.438	[1.221	1.694]	^***^	1.442	[1.202	1.730]	^***^	1.075	[0.897	1.287]		1.369	[1.006	1.864]	^**^
*living_alone*	1.068	[0.941	1.213]		1.112	[0.742	1.669]		1.155	[0.945	1.412]		1.070	[0.803	1.426]		0.872	[0.666	1.143]		0.927	[0.591	1.455]	
*non_emotional_support*	1.201	[0.967	1.493]	^*^	0.884	[0.489	1.597]		1.268	[0.912	1.764]		0.914	[0.620	1.348]		1.783	[0.998	3.186]	^**^	1.081	[0.333	3.506]	
*unhappy*	1.017	[0.820	1.260]		1.440	[0.665	3.120]		1.191	[0.861	1.648]		1.046	[0.702	1.558]		0.822	[0.534	1.267]		0.763	[0.379	1.538]	
*Southern (reference)*																								
*Northern*	0.439	[0.376	0.513]	^***^	0.343	[0.193	0.608]	^***^	0.391	[0.295	0.519]	^***^	0.358	[0.270	0.473]	^***^	0.653	[0.497	0.856]	^***^	0.480	[0.295	0.783]	^***^
*Western*	0.459	[0.404	0.522]	^***^	0.388	[0.242	0.622]	^***^	0.328	[0.265	0.406]	^***^	0.406	[0.324	0.508]	^***^	0.590	[0.468	0.742]	^***^	0.902	[0.651	1.250]	
*Central_and_Eastern*	0.862	[0.758	0.980]	^**^	1.076	[0.625	1.852]		0.801	[0.645	0.995]	^**^	0.757	[0.598	0.959]	^**^	0.919	[0.710	1.190]		1.006	[0.703	1.438]	
*Constant*	0.560	[0.457	0.686]	^***^	0.633	[0.351	1.143]	^*^	0.916	[0.698	1.201]		0.967	[0.697	1.342]		0.933	[0.646	1.348]		0.331	[0.203	0.540]	^***^

Notes: ^***^, ^**^ and ^*^ indicate significance at 1%, 5% and 10%, respectively. Source: Authors’ elaboration.

**Table 4:** Logistic estimates. Dependent variable: *smoker.*

VARIABLES	FULL SAMPLE (n = 29,337)				SILENT GENERATION (n = 2,084)				BABY BOOMERS (n = 9,086)				GENERATION X (n = 8,089)				MILLENNIALS (n = 6,906)				GENERATION Z (n = 3,172)			
	OR	95%CI			OR	95%CI			OR	95%CI			OR	95%CI			OR	95%CI			OR	95%CI		
*silent_generation*	0.182	[0.127	0.262]	^***^																				
*baby_boomers*	0.829	[0.687	1.000]	^**^																				
*generation_x*	1.471	[1.221	1.773]	^***^																				
*millennials*	1.865	[1.534	2.267]	^***^																				
*generation_z (reference)*																								
*female*	0.711	[0.643	0.786]	^***^	0.563	[0.301	1.053]	^*^	0.707	[0.581	0.860]	^***^	0.761	[0.633	0.913]	^***^	0.668	0.539	0.827]	^***^	0.734	[0.538	1.002]	^**^
*low_education (reference)*																								
*middle_education*	0.927	[0.809	1.063]		0.918	[0.409	2.061]		0.950	[0.740	1.219]		1.003	[0.745	1.351]		0.600	0.433	0.831]	^***^	1.145	[0.815	1.611]	
*high_education*	0.465	[0.395	0.547]	^***^	0.572	[0.207	1.582]		0.565	[0.397	0.805]	^***^	0.475	[0.347	0.650]	^***^	0.281	0.201	0.393]	^***^	0.663	[0.420	1.046]	^*^
*rural*	0.819	[0.737	0.910]	^***^	0.712	[0.361	1.407]		0.785	[0.630	0.978]	^**^	0.818	[0.679	0.985]	^**^	0.845	0.685	1.042]	^*^	0.829	[0.610	1.127]	
*low_income (reference)*																								
*middle_income*	1.026	[0.899	1.170]		1.099	[0.557	2.170]		1.269	[0.966	1.667]	^*^	0.834	[0.657	1.059]		0.989	0.765	1.277]		1.110	[0.744	1.657]	
*high_income*	0.881	[0.750	1.034]		1.305	[0.430	3.963]		1.176	[0.803	1.724]		0.679	[0.504	0.916]	^***^	0.910	0.685	1.209]		0.975	[0.630	1.511]	
*sah_b_vb*	1.480	[1.192	1.839]	^***^	2.492	[1.109	5.602]	^**^	1.378	[0.984	1.929]	^*^	1.414	[0.941	2.126]	^*^	1.342	0.748	2.408]		2.727	[1.184	6.277]	^**^
*disabled*	1.093	[0.957	1.248]		1.341	[0.700	2.570]		0.943	[0.741	1.199]		1.180	[0.919	1.517]		1.159	0.851	1.578]		1.198	[0.802	1.791]	
*non_social_meeting*	1.020	[0.835	1.246]		0.895	[0.455	1.758]		0.921	[0.678	1.249]		1.150	[0.807	1.639]		0.911	0.604	1.376]		0.525	[0.260	1.059]	^*^
*non_social_act*	1.015	[0.908	1.135]		1.544	[0.804	2.963]		1.330	[1.078	1.640]	^***^	1.178	[0.958	1.449]	^*^	0.783	0.637	0.961]	^**^	0.702	[0.513	0.959]	^**^
*living_alone*	1.406	[1.227	1.611]	^***^	1.391	[0.726	2.668]		1.609	[1.224	2.115]	^***^	1.131	[0.874	1.465]		1.605	1.205	2.139]	^***^	1.497	[0.971	2.308]	^*^
*non_emotional_support*	1.116	[0.865	1.440]		1.139	[0.499	2.602]		1.309	[0.904	1.897]		0.690	[0.403	1.183]		1.818	0.999	3.307]	^**^	1.295	[0.354	4.735]	
*unhappy*	1.377	[1.091	1.738]	^***^	0.940	[0.312	2.827]		0.865	[0.585	1.279]		1.823	[1.195	2.780]	^***^	1.580	0.972	2.568]	^*^	1.361	[0.694	2.669]	
*Southern (reference)*																								
*Northern*	0.677	[0.561	0.816]	^***^	2.041	[0.665	6.263]		0.978	[0.671	1.424]		0.684	[0.501	0.935]	^**^	0.613	0.440	0.852]	^***^	0.368	[0.223	0.606]	^***^
*Western*	0.780	[0.677	0.898]	^***^	1.874	[0.730	4.806]		0.883	[0.658	1.186]		0.804	[0.625	1.035]	^*^	0.748	0.577	0.970]	^**^	0.620	[0.417	0.921]	^**^
*Central_and_Eastern*	1.156	[0.985	1.357]	^*^	1.426	[0.517	3.931]		1.333	[1.006	1.767]	^**^	1.266	[0.970	1.652]	^*^	1.110	0.833	1.477]		1.012	[0.670	1.526]	
*Constant*	0.420	[0.333	0.530]	^***^	0.028	[0.010	0.081]	^***^	0.242	[0.173	0.340]	^***^	0.621	[0.459	0.841]	^***^	1.317	0.908	1.910]		0.429	[0.261	0.707]	^***^

Notes: ^***^, ^**^ and ^*^ indicate significance at 1%, 5% and 10%, respectively. Source: Authors’ elaboration.

**Table 5:** Logistic estimates. Dependent variable: *frequent_drinker.*

VARIABLES	FULL SAMPLE (n = 29,337)				SILENT GENERATION (n = 2,084)				BABY BOOMERS (n = 9,086)				GENERATION X (n = 8,089)				MILLENNIALS (n = 6,906)				GENERATION Z (n = 3,172)			
	OR	95%CI			OR	95%CI			OR	95%CI			OR	95%CI			OR	95%CI			OR	95%CI		
*silent_generation*	3.614	[2.794	4.676]	^***^																				
*baby_boomers*	3.661	[2.983	4.494]	^***^																				
*generation_x*	2.395	[1.946	2.946]	^***^																				
*millennials*	1.605	[1.288	2.000]	^***^																				
*generation_z (reference)*																								
*female*	0.289	[0.260	0.322]	^***^	0.219	[0.133	0.362]	^***^	0.294	[0.247	0.350]	^***^	0.295	[0.243	0.359]	^***^	0.264	[0.209	0.333]	^***^	0.441	[0.292	0.666]	^***^
*low_education (reference)*																								
*middle_education*	1.163	[0.992	1.364]	^*^	0.903	[0.511	1.598]		0.968	[0.753	1.244]		1.079	[0.822	1.416]		1.222	[0.790	1.890]		2.339	[1.353	4.045]	^***^
*high_education*	1.568	[1.329	1.850]	^***^	1.889	[1.063	3.358]	^**^	1.429	[1.088	1.876]	^***^	1.452	[1.102	1.912]	^***^	1.188	[0.781	1.807]		4.346	[2.476	7.628]	^***^
*rural*	1.112	[0.988	1.250]	^*^	0.936	[0.604	1.449]		1.116	[0.924	1.347]		0.972	[0.796	1.188]		1.217	[0.926	1.598]		1.618	[1.051	2.492]	^**^
*low_income (reference)*																								
*middle_income*	1.114	[0.942	1.316]		1.459	[0.890	2.393]	^*^	1.280	[1.012	1.619]	^**^	0.939	[0.714	1.236]		1.058	[0.740	1.511]		0.727	[0.427	1.240]	
*high_income*	1.355	[1.152	1.594]	^***^	1.415	[0.702	2.852]		1.429	[1.055	1.934]	^**^	1.391	[1.045	1.851]	^**^	1.344	[0.952	1.898]	^*^	0.956	[0.564	1.621]	
*sah_b_vb*	0.791	[0.623	1.004]	^**^	0.767	[0.353	1.667]		0.639	[0.437	0.937]	^**^	1.101	[0.739	1.640]		0.550	[0.233	1.298]		1.656	[0.417	6.570]	
*disabled*	0.917	[0.797	1.056]		0.635	[0.428	0.943]	^**^	0.961	[0.769	1.201]		1.008	[0.775	1.312]		0.850	[0.593	1.219]		0.721	[0.409	1.270]	
*non_social_meeting*	0.779	[0.623	0.975]	^**^	0.711	[0.308	1.637]		0.909	[0.652	1.268]		0.667	[0.462	0.964]	^**^	0.663	[0.367	1.197]		0.455	[0.119	1.734]	
*non_social_act*	0.777	[0.696	0.868]	^***^	0.917	[0.553	1.521]		0.786	[0.645	0.957]	^**^	0.868	[0.713	1.057]		0.779	[0.612	0.992]	^**^	0.479	[0.300	0.765]	^***^
*living_alone*	1.177	[1.023	1.354]	^**^	1.068	[0.641	1.780]		1.012	[0.791	1.294]		1.447	[1.092	1.917]	^***^	1.320	[0.937	1.860]	^*^	1.418	[0.855	2.350]	
*non_emotional_support*	0.939	[0.698	1.265]		0.896	[0.467	1.717]		0.940	[0.627	1.411]		1.171	[0.662	2.071]		0.920	[0.294	2.880]		0.654	[0.169	2.523]	
*unhappy*	1.034	[0.786	1.359]		1.075	[0.524	2.205]		0.762	[0.460	1.261]		1.247	[0.819	1.899]		1.062	[0.642	1.757]		1.603	[0.565	4.542]	
*Southern (reference)*																								
*Northern*	0.368	[0.300	0.451]	^***^	0.345	[0.136	0.875]	^**^	0.382	[0.273	0.535]	^***^	0.394	[0.283	0.549]	^***^	0.362	[0.238	0.552]	^***^	0.357	[0.176	0.727]	^***^
*Western*	1.086	[0.947	1.246]		1.450	[0.820	2.566]		1.186	[0.941	1.495]		1.057	[0.837	1.337]		1.059	[0.777	1.445]		0.999	[0.661	1.511]	
*Central_and_Eastern*	0.395	[0.332	0.470]	^***^	0.517	[0.275	0.972]	^**^	0.375	[0.284	0.494]	^***^	0.437	[0.316	0.604]	^***^	0.405	[0.272	0.602]	^***^	0.402	[0.231	0.701]	^***^
*Constant*	0.188	[0.147	0.240]	^***^	0.731	[0.366	1.461]		0.696	[0.517	0.939]	^**^	0.463	[0.325	0.660]	^***^	0.358	[0.221	0.582]	^***^	0.117	[0.060	0.229]	^***^

Notes: ^***^, ^**^ and ^*^ indicate significance at 1%, 5% and 10%, respectively. Source: Authors’ elaboration.

**Table 6:** Logistic estimates. Dependent variable: *obese_overweight.*

VARIABLES	FULL SAMPLE (n = 29,337)				SILENT GENERATION (n = 2,084)				BABY BOOMERS (n = 9,086)				GENERATION X (n = 8,089)				MILLENNIALS (n = 6,906)				GENERATION Z (n = 3,172)			
	OR	95%CI			OR	95%CI			OR	95%CI			OR	95%CI			OR	95%CI			OR	95%CI		
*silent_generation*	3.033	[2.423	3.797]	^***^																				
*baby_boomers*	4.704	[3.991	5.544]	^***^																				
*generation_x*	3.880	[3.274	4.599]	^***^																				
*millennials*	2.364	[1.986	2.812]	^***^																				
*generation_z (reference)*																								
*female*	0.518	[0.469	0.571]	^***^	0.739	[0.512	1.065]		0.572	[0.479	0.684]	^***^	0.478	[0.398	0.576]	^***^	0.426	[0.356	0.511]	^***^	0.613	[0.455	0.826]	^***^
*low_education (reference)*																								
*middle_education*	0.955	[0.842	1.083]		1.022	[0.680	1.535]		0.931	[0.750	1.156]		0.828	[0.641	1.071]		0.912	[0.655	1.269]		1.446	[1.027	2.035]	^**^
*high_education*	0.683	[0.593	0.786]	^***^	0.915	[0.538	1.559]		0.581	[0.457	0.739]	^***^	0.630	[0.475	0.836]	^***^	0.591	[0.418	0.835]	^***^	1.494	[0.978	2.284]	^*^
*rural*	1.094	[1.002	1.194]	^**^	1.530	[1.080	2.168]	^**^	1.148	[0.984	1.339]	^*^	1.071	[0.909	1.263]		0.992	[0.814	1.209]		1.187	[0.893	1.579]	
*low_income (reference)*																								
*middle_income*	0.975	[0.856	1.110]		1.145	[0.767	1.711]		0.959	[0.774	1.189]		1.051	[0.809	1.364]		1.018	[0.797	1.299]		0.791	[0.528	1.184]	
*high_income*	0.947	[0.817	1.098]		1.832	[1.017	3.300]	^**^	0.945	[0.724	1.234]		0.936	[0.707	1.240]		0.900	[0.686	1.180]		1.003	[0.670	1.502]	
*sah_b_vb*	1.194	[0.983	1.450]	^*^	0.956	[0.615	1.487]		0.992	[0.736	1.337]		1.456	[0.974	2.179]	^*^	1.708	[1.044	2.797]	^**^	1.351	[0.634	2.881]	
*disabled*	1.229	[1.097	1.377]	^***^	1.826	[1.296	2.574]	^***^	1.204	[0.992	1.460]	^*^	1.210	[0.966	1.517]	^*^	1.058	[0.820	1.365]		1.188	[0.841	1.677]	
*non_social_meeting*	1.108	[0.942	1.303]		0.723	[0.433	1.208]		1.115	[0.872	1.424]		0.995	[0.726	1.363]		1.485	[1.030	2.141]	^**^	1.205	[0.634	2.291]	
*non_social_act*	1.115	[1.013	1.227]	^**^	1.085	[0.765	1.540]		0.946	[0.797	1.124]		1.180	[0.980	1.420]	^*^	1.145	[0.960	1.366]	^*^	1.219	[0.919	1.616]	
*living_alone*	0.841	[0.739	0.959]	^***^	0.861	[0.588	1.259]		0.804	[0.655	0.988]	^**^	0.944	[0.732	1.217]		0.597	[0.443	0.803]	^***^	1.246	[0.797	1.948]	
*non_emotional_support*	0.920	[0.731	1.158]		0.893	[0.509	1.566]		1.049	[0.757	1.455]		0.591	[0.366	0.954]	^**^	1.144	[0.621	2.108]		1.339	[0.523	3.428]	
*unhappy*	0.822	[0.673	1.003]	^**^	0.875	[0.475	1.611]		0.877	[0.624	1.231]		0.692	[0.476	1.006]	^**^	0.866	[0.554	1.354]		0.833	[0.386	1.796]	
*Southern (reference)*																								
*Northern*	1.158	[1.010	1.328]	^**^	0.673	[0.389	1.164]		0.913	[0.700	1.191]		1.420	[1.072	1.883]	^**^	1.591	[1.203	2.105]	^***^	0.910	[0.578	1.433]	
*Western*	0.969	[0.871	1.079]		0.468	[0.288	0.759]	^***^	0.798	[0.652	0.976]	^**^	1.135	[0.918	1.405]		1.115	[0.886	1.403]		1.105	[0.783	1.561]	
*Central_and_Eastern*	1.200	[1.059	1.360]	^***^	0.588	[0.351	0.984]	^**^	1.345	[1.074	1.683]	^***^	1.447	[1.130	1.852]	^***^	1.164	[0.885	1.531]		0.884	[0.610	1.281]	
*Constant*	0.576	[0.474	0.701]	^***^	1.554	[0.834	2.894]		3.236	[2.411	4.344]	^***^	2.174	[1.505	3.141]	^***^	1.557	[1.099	2.205]	^***^	0.352	[0.197	0.629]	^***^

Notes: ^***^, ^**^ and ^*^ indicate significance at 1%, 5% and 10%, respectively. Source: Authors’ elaboration.

Firstly, in Table 3, it can be observed that older generations are at greater risk for physical inactivity. In fact, the OR increases with age. Regarding socio-economic factors, being a female increase the likelihood of being inactive (being no significant for the Baby Boomers, Generation X, and Millennials) whereas education appears, when significant, to be “protective”. Rural areas do not appear to have generally robust significant results and seems to be harmful to Silent Generation and Baby Boomers. No extremely statistically significant results are obtained for income but, when significant, it appears to be caring. As for health factors, higher inactivity would be associated with those with bad or very bad self-assessed health and with disabilities. This factor is less relevant for the youngest. Besides, regarding macro-region, being in the Southern would increase the likelihood of being inactive. As for social isolation factors, *non_social_meeting* appears to be the variable consistently associated with physical inactivity across all generations followed by *non_social_act* (except for Millennials). Besides, *non_emotional_support* confirms weak or age-specific effect, when significantly associated with greater inactivity (full sample and Millennials). Finally, *living_alone* or *unhappy* do not show a clear significant result.

Secondly, in Table 4, it is determined that older generations (both Silent Generation and Baby Boomers) are less likely to smoke whereas Generation X and Millennials are more likely to smoke (Generation Z is considered as reference). In other words, the results suggest the existence of a generational divide in smoking habits within the analyzed sample. Turning to socio-economic factors it is obtained that being a female reduce the likelihood of being smoker. Regarding education, when significant, appears to be “protective” (being education not significant for the eldest population). Living in a rural area, when significant (full sample, Baby Boomers, Generation X, and Millennials), reduce also the likelihood of being smoker. As for income, higher income lowers the likelihood of being smoker. This pattern is clearer and significant for Generation X. Turning to health factors, the worse health status, the higher likelihood of being smoker (not affecting this health variables for the Millennials). Not being disability significant in any case. Besides, it is determined, when significant, that people from Northern and Western regions are less likely to smoke. Concerning social isolation factors, on the one hand, slightly significant results are obtained for *non_social_meeting* or *non_emotional_support* (just for Generation Z and Millennials, respectively). On the other hand, living alone or feeling unhappy are the variables that appear to be more consistent with the likelihood of being a smoker. Especially, from Baby Boomers and Generation X though the younger population (Millennials). However, the effect of non-participating in social activities is less stable since it increases the likelihood of being a smoker in Baby Boomers, but it is negative for Millennials and Generation Z (the youngest), suggesting a trend changed in the connection between socialization and smoking.

Thirdly, in Table 5, results for *frequent_drinker* are provided. It can clearly observe that the older the generation, the greater the risk for frequent drinking. With respect to socio-economic factors, it is claimed again that women tend to have healthier lifestyles and/or lower health risk. Here, associated with alcohol consumption. However, the higher the educational level the higher the likelihood of being frequent drinker (this variable is not significant for Millennials). In the same line, the higher the income or living in rural areas the higher the likelihood of being frequent drinker. In terms of health factors, when significant, the worse health factors the lower the likelihood of being frequent drinker. These factors are especially significant for the Silent Generation and Baby Boomers (the older ones, not mattering to the young ones). Regarding macro-areas, both Northern and Central and Eastern ones appear to provide a lower likelihood of being frequent drinkers. As it is related to social-isolation factors, on the one hand, non-social meetings is quite consistent associated with a lower likelihood of frequent drinking (at least for the full sample and Generation X). Results are similar for non-social activities, here for the full sample, Baby Boomers and the younger (both the Millenials and Generation Z). However, living alone increases the likelihood of being a frequent drinker (principally for Generation X, and Millennials). Moreover, non-emotional support and unhappy are not significant when explaining this risk factor.

Lastly, Table 6 allocates the findings for *obese_overweight* health risk factor. It is highlighted that being in a generation prior to Generation Z would have a higher likelihood of being obese or overweight. Baby Boomers present the highest risk. About socio-economic factors, being a female, again, reduce the risk. In the same line, higher education would affect for all the generation except the Silent Generation for which this variable is not significant or for the youngest where the reverse effect is found. Living in a rural area would be detrimental for this dependent variable (mainly for the elder). Non-statistically significant results are obtained for income (the exception in high income for the Silent Generation). In relation to health factors, in general, the worse they are the worse result for BMI (but not being significant for Generation Z). As for macro-regions, Western ones, when significant, provide the less likelihood of being obese or overweight. When it comes to social-isolation factors, lack of socialization provides a higher likelihood of obesity and/or overweight in younger generations (Millennials) while living alone would reduce this risk. Emotional support and unhappiness variables would be thought-provoking for Generation X.

To sum up, this analysis of the factors among our four dependent variables has reveal general patterns that could identify higher risk variables/factors and/or different behaviors that are essential to comprehensively address the determinants of health outcomes and so establish efficient and effective public health policies.

In general terms, results have determined different conducts associated to gender (being a female would be generally protective, except for physical activity). Besides, higher education appears to be also a protective factor (being the frequent consumption of alcohol the exception), perhaps due to differences in leisure and/or lifestyles of these individuals. However, living in rural areas has provided heterogeneous effects. The explanation could be related to cultural differences, access to healthcare services, etc... The same applies to income. Are higher income individuals associated with a sedentary lifestyle pattern with higher access to caloric food and/or alcoholic beverages? The empirical findings suggest that. Additionally, it has been highlighted that both limitations and medical conditions (self-rated) affect health risk factors. Regarding social-isolation factors, low socialization, living alone or low emotional support appear to be important. So, the social environment is confirmed to be a key element when analysis healthy habits. By regions, many dissimilarities have been seen. This may be associated with regional and cultural differences in Europe.

In addition, when focusing on generations, it can be stressed that differences have appeared through Tables 3–6, indicating that social norms and consciousness about health have been changing over time. Then, one could say that Generation X maintains an intermediate level on these behaviors. That is, it is a transition group in between traditional and modern lifestyles.

All in all, in our estimations we had pooled data from 24 countries and controlled for four macro-regions. To handle with possible country-level variance, as robustness checks, we had re-estimated the full sample model using multilevel logistic regression models accounting for the country level. All these results, again, are reported as odds ratios with 95% confidence intervals. The corresponding table with these robustness checks is presented in the Supplementary material (S1 Table). Importantly, the robustness approached yield results that are quite consistent with those reported in the main analysis (first columns of Tables 3–6).

## Discussion

This study sets out to examine the association between several dimensions of social isolation (not loneliness) and key health-related behaviors (physical inactivity, smoke and frequent drinking consumption, and overweight/obesity throughout BMI) across five generational cohorts (Silent Generation, Baby Boomers, Generation X, Millennials, and Generation Z) in Europe. Drawing on data from the European Social Survey, the findings provide valuable insights into how distinct forms of isolation (lack of social meetings, non-participation in activities, living alone, lack of emotional support, unhappy) influence lifestyle risks, and how these associations vary by age group. Recent literature has proposed new approaches to measure social isolation (“difficulty going out” and lack of participation) as risk indicators for hikikomori-like syndromes in Europe, which is consistent with our operationalization of isolation and further validates its association with behavioral health risks.

Our findings indicate that although social isolation is less common in early adulthood, its health results may be especially pronounced in younger generations. In our analysis, socially disconnected young adults showed strong associations with unfavorable health behaviors, echoing prior suggestions that health risks of isolation accumulate over years. This result expands on research focused on young, middle and old age, with younger individuals at growing risk [13,48].

Consistent with previous studies, we found robust associations between social isolation and physical inactivity. All our generations were more likely to be physically inactive if they did not have frequent social meetings. This pattern is consistent with previous literature, showing that isolated young people had a higher prevalence of physical inactivity as they exercised fewer days per week [49]. This may mean that the lack of social connections removes important sources of reinforcement, motivation, or opportunities for an active lifestyle [13]; while socially integrated individuals receive more encouragement and support to exercise [50]. These findings may reflect that social disconnection during early adulthood disrupts the establishment of health-promoting habits, such as group-based physical activity or peer accountability regarding substance use.

The relationship between social isolation and smoking in our study suggests that living alone or declaring being unhappy are associated with smoking across all generations (except for the Silent Generation), while not participating in social activities is associated with a lower likelihood of smoking for the youngest (Millennials and Generation Z). This is in line with several studies [50]. Dyal and Valente [51], in a meta-review, found half of studies reported no loneliness–smoking association while the others found only a small effect. Other studies have also reported positive isolation–smoking correlations [52]. This may suggest the influence of social isolation on smoking may depend on life stage and social norms. Thus, social networks may favor the initiation of use in young people and isolation may act as a protective factor, while tobacco may be used as a coping mechanism for loneliness in older adults and isolation may increase use.

With respect to alcohol use, our findings reveal that isolated individuals (with limited participation in social meetings or activities) are less likely to consume alcohol frequently. In fact, empirical evidence suggests that isolation can be associated with lower heavy alcohol use, particularly in social drinking cultures. Jensen et al. [50] indicated that lonely adolescents and young adults had significantly lower odds of high-frequency or high-quantity alcohol consumption compared to non-lonely individuals, as suggested by Arpin et al. [53]. Thus, social isolation might protect against certain social forms of alcohol misuse. However, this does not imply that isolated individuals may resort to solitary drinking as a way of coping with stress, depression or anxiety [54]. On the one hand, social isolation may diminish social drinking by removing positive peer influences that encourage moderation. On the other hand, the cost of social support may heighten stress and promote solitary drinking among susceptible individuals. Some research examines how loneliness differentially affects what people drink, how they drink (socially or alone), and who is most at risk, showing that young men’s drinking is more affected by social isolation than women’s [55].

According to BMI, we found that younger generations of isolated individuals (with limited participation in social meetings or activities) were significantly more likely to report higher BMI. This may suggest that early social disengagement is associated with weight gain through reduced physical activity and poor diet. Some authors have pointed out that this association can be bidirectional. Isolation can encourage sedentary lifestyles and emotional eating, leading to weight gain [37,56]; in turn, obesity can induce social isolation due to stigma or low self-esteem [57].

This study presents several strengths. First, it draws on a large, harmonized dataset from the ESS, enabling cross-national comparisons and age cohort analysis across diverse European populations. The use of different types of social isolation (non-social meetings, non-social activities, living alone, non-emotional support, unhappy) allows us to compare different ways in which individuals may feel or be isolated, which reinforces the internal validity of our results. Moreover, the generational approach (Silent Generation, Baby Boomers, Generation X, Millennials, Generation Z), provides insights into how the relationship between social isolation and lifestyle factors evolves across the life course, with a particular emphasis on young adults, a population underrepresented in isolation research.

However, this study also presents some limitations. The cross-sectional nature of the ESS data precludes causal inference. It remains unclear whether social isolation precedes the development of unhealthy behaviors, or if the relationship is bidirectional. Besides, although multicollinearity was not initially detected among the variables included in the models, residual confounding related to age and other unmeasured factors cannot be completely ruled out and should be considered when interpreting the observed results. Additionally, the reliance on self-reported data may introduce biases, particularly in sensitive behaviors such as smoking or alcohol consumption. In addition, BMI was calculated from self-reported height and weight, which may lead to measurement error, as respondents often underreport weight or overreport height. Moreover, social isolation was based on frequency of social contact and participation, which may not fully capture subjective experiences of isolation. It was not possible to analyze how social isolation, and loneliness may differ in their associations with health-related factors as the loneliness variable was not available in the wave considered in this study. Missing values were handled by excluding observations with ESS non-substantive responses, including “Refusal”, “Don’t know”, and “No answer”, in any of the variables included in the models. In addition, several variables were dichotomized to facilitate interpretation and comparability across health-risk outcomes. However, dichotomization entails a loss of information and statistical power, particularly for variables originally measured on broader scales, such as happiness, and for continuous measures such as BMI.

Future research should disaggregate the observed patterns by gender, examining whether the associations between social isolation and lifestyle factors differ for women and men. Such analyses may reveal gendered mechanisms of vulnerability or resilience and enable more targeted and equitable public health interventions.

## Conclusions

Social isolation has emerged as a critical determinant of health, particularly in the context of lifestyle-related behaviors. Across generations, the experience and health consequences of isolation differ, shaped by digital literacy, socio-economic conditions, cultural attitudes, and historical events. While older adults have traditionally been the focus of isolation-related research, this study highlights that younger generations, particularly Millennials and Generation Z, are also highly vulnerable to the effects of social isolation.

Our findings reinforce the growing evidence that social integration is a fundamental social determinant of health throughout life. Public health data show that young adults report some of the highest rates of loneliness, higher than those of older age groups, highlighting the urgency of addressing social isolation in the young [58]. The strong associations observed between social isolation and behaviors such as physical inactivity, poor diet, and excess weight in young people point to early adulthood as a key intervention point.

Promoting social relations may serve as a powerful lever to improve health-related behaviors across generations. Community-based or peer-supported interventions that facilitate group exercise, social participation, and access to nutritional guidance could reduce isolation and mitigate obesity risk, especially in socially isolated young people [59]. Tackling both sides of this cycle is essential to prevent long-term health consequences. To that end, several public policy actions are warranted. Community interventions specifically targeting lonely youth should be developed, for example through neighborhood programs that integrate isolated individuals into social, cultural, or physical activities. Public health strategies must incorporate the social dimension into routine lifestyle monitoring and health promotion, recognizing that social isolation is both a risk factor and a modifiable target.

This study adds to the literature by demonstrating that the health links of isolation are not confined to the elderly and may, in some cases, be more acute among younger individuals. For these reasons, public health strategies should broaden their scope to include socially isolated youth, integrating social connection into health promotion and disease prevention.

## Supporting information

S1 Table Multi-level model (hierarchical logistic regression) at the country level (full sample, n = 29,337). (DOCX)
