# Supplementary material for: Social isolation and health risks: Do the younger generations behave differently? Findings from the European Social Survey (Round 11 – 2023/2024)
Source: PLoS One. 2026 Sep 10;21(9):e0357725. doi: 10.1371/journal.pone.0357725 (PMC13561355; doi:10.1371/journal.pone.0357725)
Supplement: Supplementary file 1 [file pone.0357725.s001.docx]

**Table S1. Multi-level model (hierarchical logistic regression) at the country level (full sample, n = 29,337).**

| **VARIABLES** | ***physical_inacvity*** | | | | ***smoker*** | | | | | ***frequent_drinker*** | | | | | ***obese_overweight*** | | | | |  |
| --- | --- | --- | --- | --- | --- | --- | --- | --- | --- | --- | --- | --- | --- | --- | --- | --- | --- | --- | --- | --- |
|  | **OR** | **95%CI** | |  | | **OR** | **95%CI** | |  | | **OR** | **95%CI** | |  | | **OR** | **95%CI** | |  | |
| *silent_generation* | 2.560 | [2.253 | 2.908] | ^***^ | | 0.151 | [0.122 | 0.187] | ^***^ | | 3.103 | [2.639 | 3.648] | ^***^ | | 3.189 | [2.816 | 3.612] | ^***^ | |
| *baby_boomers* | 1.638 | [1.495 | 1.795] | ^***^ | | 0.696 | [0.627 | 0.774] | ^***^ | | 3.414 | [3.014 | 3.867] | ^***^ | | 4.545 | [4.146 | 4.983] | ^***^ | |
| *generation_x* | 1.560 | [1.423 | 1.710] | ^***^ | | 1.375 | [1.242 | 1.522] | ^***^ | | 2.435 | [2.146 | 2.762] | ^***^ | | 4.103 | [3.741 | 4.499] | ^***^ | |
| *millennials* | 1.467 | [1.334 | 1.612] | ^***^ | | 1.578 | [1.422 | 1.752] | ^***^ | | 1.580 | [1.386 | 1.802] | ^***^ | | 2.449 | [2.230 | 2.690] | ^***^ | |
| *generation_z (reference)* |  |  |  |  | |  |  |  |  | |  |  |  |  | |  |  |  |  | |
| *female* | 1.108 | [1.054 | 1.165] | ^***^ | | 0.643 | [0.607 | 0.682] | ^***^ | | 0.290 | [0.272 | 0.308] | ^***^ | | 0.515 | [0.490 | 0.541] | ^***^ | |
| *low_education (reference)* |  |  |  |  | |  |  |  |  | |  |  |  |  | |  |  |  |  | |
| *middle_education* | 0.836 | [0.779 | 0.897] | ^***^ | | 0.929 | [0.857 | 1.006] | ^*^ | | 1.156 | [1.057 | 1.263] | ^***^ | | 0.967 | [0.901 | 1.037] |  | |
| *high_education* | 0.651 | [0.602 | 0.704] | ^***^ | | 0.513 | [0.467 | 0.563] | ^***^ | | 1.484 | [1.348 | 1.633] | ^***^ | | 0.717 | [0.664 | 0.774] | ^***^ | |
| *rural* | 1.000 | [0.949 | 1.053] |  | | 0.868 | 0.817 | 0.922] | ^***^ | | 1.069 | [1.004 | 1.138] | ^**^ | | 1.123 | [1.067 | 1.182] | ^***^ | |
| *low_income (reference)* |  |  |  |  | |  |  |  |  | |  |  |  |  | |  |  |  |  | |
| *middle_income* | 1.009 | [0.944 | 1.080] | ^**^ | | 0.961 | [0.890 | 1.038] |  | | 1.047 | [0.963 | 1.138] |  | | 0.999 | [0.935 | 1.068] |  | |
| *high_income* | 1.023 | [0.944 | 1.109] |  | | 0.812 | [0.739 | 0.892] | ^***^ | | 1.260 | [1.141 | 1.390] | ^***^ | | 0.991 | [0.915 | 1.072] |  | |
| *sah_b_vb* | 1.953 | [1.747 | 2.183] | ^***^ | | 1.222 | [1.080 | 1.383] | ^***^ | | 0.689 | [0.597 | 0.794] | ^***^ | | 1.062 | [0.953 | 1.184] | ^*^ | |
| *disabled* | 1.431 | [1.344 | 1.523] | ^***^ | | 1.180 | [1.094 | 1.272] | ^***^ | | 0.964 | [0.893 | 1.041] |  | | 1.301 | [1.222 | 1.385] | ^***^ | |
| *non_social_meeting* | 1.445 | [1.321 | 1.581] | ^***^ | | 0.900 | [0.812 | 0.997] | ^**^ | | 0.794 | [0.702 | 0.898] | ^***^ | | 1.039 | [0.951 | 1.136] |  | |
| *non_social_act* | 1.415 | [1.341 | 1.494] | ^***^ | | 1.063 | [0.998 | 1.133] | ^*^ | | 0.785 | [0.734 | 0.839] | ^***^ | | 1.051 | [0.997 | 1.109] | ^*^ | |
| *living_alone* | 0.969 | [0.906 | 1.037] |  | | 1.295 | [1.199 | 1.398] | ^***^ | | 1.075 | [0.992 | 1.165] | ^*^ | | 0.876 | [0.820 | 0.936] | ^***^ | |
| *non_emotional_support* | 1.125 | [0.988 | 1.281] | ^*^ | | 1.033 | [0.895 | 1.193] |  | | 1.089 | [0.930 | 1.275] |  | | 0.988 | [0.870 | 1.123] |  | |
| *unhappy* | 1.066 | [0.949 | 1.198] |  | | 1.292 | [1.143 | 1.461] | ^***^ | | 1.088 | [0.939 | 1.262] |  | | 0.949 | [0.847 | 1.064 | ^**^ | |
| *Southern (reference)* |  |  |  |  | |  |  |  |  | |  |  |  |  | |  |  |  |  | |
| *Northern* | 0.343 | [0.240 | 0.490] | ^***^ | | 0.427 | [0.260 | 0.701] | ^***^ | | 0.463 | [0.294 | 0.729] | ^***^ | | 1.217 | [0.964 | 1.535] | ^*^ | |
| *Western* | 0.423 | [0.307 | 0.582] | ^***^ | | 0.679 | [0.436 | 1.058] | ^*^ | | 1.314 | [0.876 | 1.971] |  | | 0.885 | [0.719 | 1.090] |  | |
| *Central_and_Eastern* | 0.699 | [0.497 | 0.982] | ^**^ | | 1.098 | [0.686 | 1.756] | ^*^ | | 0.540 | [0.350 | 0.833] | ^***^ | | 1.235 | [0.988 | 1.543] | ^**^ | |
| *Constant* | 0.745 | [0.567 | 0.978] | ^**^ | | 0.547 | [0.379 | 0.790] | ^***^ | | 0.176 | [0.124 | 0.250] | ^***^ | | 0.548 | [0.451 | 0.665] | ^***^ | |

Notes: ^***^, ^**^ and ^*^ indicate significance at 1%, 5% and 10%, respectively. Source: Authors’ elaboration.
